# Draft Genome Sequences of Four Clinical Legionella pneumophila Isolates from Ontario, Canada

**DOI:** 10.1128/genomeA.00295-18

**Published:** 2018-04-12

**Authors:** Alexander Fortuna, Ricardo Ramnarine, Aimin Li, Nahuel Fittipaldi, Christine Frantz, Gustavo V. Mallo

**Affiliations:** a Public Health Ontario, Toronto, Canada; b Department of Laboratory Medicine and Pathobiology, University of Toronto, Toronto, Canada

## Abstract

Legionella pneumophila outbreak investigations require the development of reliable typing methods to better understand the genetic relationships of the isolates involved. Here, we report the draft genome sequences of four clinical Legionella pneumophila isolates obtained between 2000 and 2012 in Ontario, Canada.

## GENOME ANNOUNCEMENT

Legionella pneumophila is a bacterial pathogen responsible for Legionnaire's disease, a severe pneumonia-like illness with a high mortality rate (1). L. pneumophila may be found in natural and man-made environments and is transmitted through the inhalation of aerosolized water droplets containing the bacterium (2). The L. pneumophila isolates in this study were collected from patients in Ontario, Canada, between 2000 and 2012.

Whole-genome sequencing (WGS) is transforming the way infectious disease bacterial research is performed, providing increased resolution in outbreak scenarios (3). As part of an outbreak investigation, it is important to develop reliable methods for the typing of L. pneumophila isolates. The draft genome sequences from this study (of isolates LG57, LG59, LG61, and LG63) will be instrumental in developing new strategies for outbreak investigations in Ontario.

Genomic DNA was extracted from cultured L. pneumophila isolates using the automated easyMAG extraction system (bioMérieux Canada, Inc., Canada) and then quantified using a Qubit spectrophotometer (Thermo Fisher Scientific, USA). DNA sequencing libraries were prepared using the Nextera XT kit (Illumina, USA). Individually tagged libraries were checked on an Agilent 2100 Bioanalyzer for library quality and sequenced as a part of a flow cell using Illumina V2 chemistry with 2 × 150-bp paired-end reads in a MiSeq platform. Quality control was performed using FastQC (4), and low-quality reads were removed prior to assembly with Sickle (quality score, 30; minimum length, 50) (5). Reads were *de novo* assembled using SPAdes version 3.9.1, with k-mer lengths of 21, 33, 55, and 77 (6). The resulting contigs (>1,000 bp) were outputted to Pilon version 1.22 to correct single-nucleotide polymorphisms (SNPs) and indels (7). The resulting contigs were then ordered against a closely related complete L. pneumophila genome with Mauve aligner (8). Open reading frame identification and genome annotation were performed using NCBI Prokaryotic Genome Annotation Pipeline (PGAP) version 4.4 (9). Genome quality, size, and G+C content were estimated using Quast version 4.6.2 (10). For the four assemblies, the genome size and G+C contents varied from 3.43 to 3.59 Mb and 38.2 to 38.3%, respectively (Table 1).

**TABLE 1:** Summary of statistics for L. pneumophila draft genome assemblies

Isolate	Type	Yr	Sequence type	No. of contigs	G+C content (%)	Size (bp)	*N*_50_ (bp)	No. of coding genes	Accession no.
LG57	Chromosome	2005	222	49	38.24	3,590,292	163,641	3,172	PQXA00000000
LG59	Chromosome	2012	37	21	38.3	3,475,634	300,825	3,087	PQWZ00000000
LG61	Chromosome	2000	154	21	38.19	3,429,439	289,473	2,966	PQWY00000000
LG63	Chromosome	2001	1	41	38.27	3,447,361	175,373	3,017	PQWX00000000
pLG57	Plasmid	2005		1	37.36	76,262		81	PQXA01000049

Separately, short reads were assembled using plasmidSPAdes (11) to identify potential plasmids. We identified a plasmid contig in the LG57 strain. For confirmation, a BAM file including all paired-end reads and the SPAdes assembly graphs were submitted to Recycler (12), confirming a 76,262-bp plasmid with high sequence similarity (97%) to a 31.9-kb fragment of the L. pneumophila subsp. pneumophila strain Lorraine plasmid pLELO (GenBank accession no. FQ958212), which we termed pLG57.

### Accession number(s).

This whole-genome project has been deposited at DDBJ/EMBL/GenBank under the accession numbers listed in Table 1. The versions described in this paper are the first versions.
